# Evaluation of Nutraceutical Potential of *Carduus marianus* : Antioxidant and Hepatoprotective Effects in Paracetamol‐Induced Hepatotoxicity and GC–MS Analysis

**DOI:** 10.1002/fsn3.70474

**Published:** 2025-07-18

**Authors:** Mehreen Nasir, Faisal Gulzar, Sajida Jamil, Irfan Anjum, Abdul Malik, Suhail Akhtar, Nouman Ali

**Affiliations:** ^1^ Department of Pharmacology The University of Lahore Lahore Pakistan; ^2^ Rai Foundation Pharmacy College Sargodha Pakistan; ^3^ Department of Basic Medical Sciences, Shifa College of Pharmaceutical Sciences Shifa Tameer‐e‐Millat University Islamabad Pakistan; ^4^ Department of Pharmaceutics, College of Pharmacy King Saud University Riyadh Saudi Arabia; ^5^ Department of Biochemistry A.T. Still University of Health Sciences Kirksville Missouri USA; ^6^ Department of Biotechnology University of Central Punjab Lahore Pakistan

**Keywords:** *Carduus marianus*, GC–MS, hepatoprotective, inflammation, oxidative stress, paracetamol

## Abstract

This study evaluates the hepatoprotective effects of *Carduus marianus* in a paracetamol‐induced hepatotoxicity model using Wistar rats. Wistar rats were divided into six groups and treated with varying doses (100, 200, and 300 mg/kg) of *C. marianus* 12× aqueous solution after hepatotoxicity induction. Serum biomarkers for liver function (ALT and AST), lipid profiles, renal indicators, oxidative stress markers, and inflammatory mediators were measured. Phytochemical screening revealed alkaloids, glycosides, carbohydrates, saponins, phenols, and flavonoids, with oleic acid as the major component. Gas chromatography–mass spectrometry (GC–MS) identified oleic acid as the predominant constituent (62.96%). Treatment significantly improved liver biomarkers, lipid profiles, and renal function (*p* < 0.001) across all doses, alongside notable reductions in oxidative stress markers and inflammatory mediators (*p* < 0.001). These results highlight the potential of *C. marianus* as a promising therapeutic agent for managing liver health.

## Graphical abstract

This study evaluates the hepatoprotective potential of *Carduus marianus* aqueous extract in male Wistar rats subjected to paracetamol‐induced hepatotoxicity. Paracetamol metabolism via hepatic cytochrome P450 enzymes generates NAPQI, triggering oxidative stress, inflammation, and vascular disturbances, culminating in hepatic necrosis. Pre‐treatment with *C. marianus* for 21 days mitigated these effects by inhibiting NAPQI‐induced pathways, as evidenced by reduced oxidative and inflammatory markers and preserved hepatic histoarchitecture. These findings suggest *C. marianus* as a promising natural agent against drug‐induced liver injury.

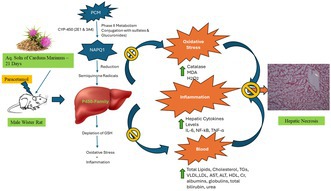

## 1 Introduction

The liver is the primary organ responsible for breaking down and metabolizing nutrients as well as pharmaceutical compounds. When liver function is compromised, it can lead to hepatocyte death and adversely affect multiple organs and systems throughout the body, as the liver plays a central role in maintaining systemic health (Abdelghffar et al. 2022). Despite recent developments in treatment, liver diseases continue to pose a severe threat to global health (Taamalli et al. 2020). About two‐thirds of all liver‐related deaths occur in men, and liver disease causes 2 million deaths a year, or 4% of all deaths (1 of every 25 deaths globally). Acute hepatitis is responsible for a lesser percentage of mortality, with cirrhosis and hepatocellular carcinoma complications accounting for the majority of deaths (Devarbhavi et al. 2023). The liver is highly susceptible to toxicity because it plays a crucial role in the metabolism of various chemicals, as well as the transport and elimination of xenobiotics. Hepatotoxicity is typically unique and involves a combination of differences in genetics, exposures to the environment, lifestyle factors, and preexisting clinical disorders. However, it is believed that the exchange of harmful foreign chemicals to ROS (reactive oxygen species) through a metabolic route, which causes cellular harm and oxidative stress, may influence the pathogenesis of chemically caused liver injury (Nasir et al. 2023). ROS, which are produced during metabolic processes as well as the biotransformation of xenobiotics, are especially harmful to the liver. Oxidative stress, a result of redox balance disruption, alters inflammatory pathways, impacts liver function, and exacerbates illness (Allameh et al. 2023).

Liver diseases account for 2 million deaths globally, representing 4% of all fatalities, with approximately two‐thirds occurring among males. Cirrhosis and hepatocellular carcinoma are the primary causes of mortality rather than acute hepatitis. The most frequent risk factors for cirrhosis include alcoholism, non‐alcoholic fatty liver disease (NAFLD), and viral hepatitis. Although medication‐induced liver damage accounts significantly for acute hepatitis, viral hepatitis remains predominant (Real et al. 2019). Obesity affects over 2 billion adults, with over 400 million people diagnosed with diabetes worldwide, predisposing individuals to NAFLD and hepatocellular carcinoma. Although liver damage induced by medication contributes significantly to acute hepatitis cases, viral hepatitis remains predominant. Regardless of the cause—whether it be medication, alcoholism, nonalcoholic factors, or viral infections—ROS play a significant role in liver damage (Asrani et al. 2019). ROS generated by various exogenous and endogenous factors induce oxidative stress, damaging cellular components like DNA, proteins, and lipids (Forni et al. 2019).

Phytochemicals derived from medicinal plants serve as valuable sources of bioactive compounds for the development of therapeutic agents targeting a wide range of diseases. These naturally occurring substances have garnered significant attention for their diverse health‐promoting properties, with over 10,000 phytochemicals identified to date. Their structural diversity determines a broad spectrum of physiological effects, most notably antioxidant activity. Diets rich in phytochemicals are widely recommended for their protective role against oxidative stress‐induced cellular damage, thereby reducing the risk of chronic conditions such as cancer, cardiovascular disease, and diabetes (Gonfa et al. 2023). In particular, plant‐derived secondary metabolites and antioxidants exhibit considerable therapeutic potential in the prevention and management of human diseases, highlighting their importance in both traditional medicine and modern drug discovery (Park 2023). These plant‐derived compounds possess a spectrum of beneficial effects, including wound healing, anti‐inflammatory, antidepressant, antidiabetic, and antimicrobial properties. Plant resilience to environmental stresses, facilitated by the synthesis of phytochemicals, underscores their importance in both plant survival and human health (Asaduzzaman and Asao 2018). Teas or extracts of natural materials are used in natural medical systems such as Ayurveda and traditional Chinese medicine to treat or cure a variety of illnesses. As antioxidants, phytochemicals in plants help protect against inflammation and illness. Numerous phytochemicals present in a wide variety of foods, such as resveratrol (RES), quercetin (QUE), curcumin (CUR), piperine (PIP), epigallocatechin gallate (EGCG), and gingerol (GIN), have been studied for their potential therapeutic benefits. These products may reduce inflammation because they have anti‐inflammatory qualities (Adetuyi et al. 2021).

An ethnobotanical approach to drug discovery has gained prominence, leveraging traditional knowledge to identify novel phytochemicals and therapeutic agents (Devarbhavi et al. 2023; Howes and Simmonds 2014). Ethnobotany, the study of human‐plant interactions, holds promise for uncovering novel phytochemicals with medicinal properties (Majumder et al. 2020). Over the past 20 years, a lot of research has been done on bioactive ingredients in food, especially phytochemicals with antioxidant properties. However, research on antioxidants has experienced major paradigm shifts as new analytical and molecular biology tools have been developed. This resulted in discoveries on phytochemicals' anti‐inflammatory qualities and modulatory impacts on cell signaling (Xiao et al. 2022).

Despite advancements in modern medicine, effective treatments for hepatotoxicity remain limited, necessitating continued research into novel therapeutic options with minimal side effects. Plants have long been recognized as a rich source of bioactive compounds, with numerous phytochemicals demonstrating hepatoprotective effects. Among these, *Silybum marianum* (milk thistle) has garnered attention for its potential hepatoprotective properties.

This study aims to elucidate the hepatoprotective effects by evaluating the phytochemical composition and antioxidant capacity of *Carduus marianus* seeds aq. extract (milk thistle) through *in vitro* phytochemical and GC–MS analysis. In addition, the hepatoprotective and anti‐inflammatory potential of *C. marianus* 12× aqueous solution was assessed in a rat model of paracetamol‐induced hepatotoxicity.

## 2 Methods and Materials

### 2.1 Plant Material

A voucher specimen of *C. marianus* (Asteraceae) has been deposited in the herbarium of Faculty of Pharmacy at the University of Lahore, Lahore, under voucher number 2945‐23 for reference. *C. marianus* is a biennial plant, and its seeds usually ripen from August to October. The seeds were obtained from a local market in October, washed with distilled water, and then crushed into powder after tray drying and cleaning.

### 2.2 Chemicals and Analytical Kits

Paracetamol and formalin of analytical grade were obtained from Sigma Aldrich and Pharmagen Limited, respectively, while distilled water was sourced from the research laboratory of Faculty of Pharmacy, the University of Lahore, Lahore. Reagents for phytochemical analysis were procured from Lahore Chemicals & Pharmaceutical Works (Pvt) Ltd. Analytical kits for estimating catalase (CAT) activity (Catalogue No: A007‐1‐1), hydrogen peroxide (H_2_O_2_) (Catalogue No: A064‐1‐1), malonaldehyde (MDA) (Catalogue No: A003‐1‐1), TNF‐α (Catalogue No: H052‐1‐2), NF‐kB (Catalogue No: H202‐1‐1), and IL‐6 (Catalogue No: H007‐1‐1) were purchased from Nanjing Jiancheng Bioengineering Institute, China. Liver function tests (LFTS), renal function tests (RFTS), lipid profile, and white blood cell (WBC) count were analyzed using a Beckman Coulter Model AU480.

### 2.3 Animals

Male Wistar rats weighing 150 ± 10 g were obtained from the Department of Pharmacology, Faculty of Pharmacy, The University of Lahore. Rats were housed in the university's animal facility under standard conditions with *ad libitum* access to food and water; temperature was maintained within 25°C ± 5°C and RH 55%–60% while maintaining a 12‐h light and dark cycle. Rats were fed with food at different intervals and were given access to drinking water to get acclimatized to the faculty animal house to ensure physiological, psychological, and nutritional stabilization before experimentation. All experimental procedures were approved by the Institutional Research Ethics Committee (IREC) of the University of Lahore, with Certification No. IREC‐2023‐48.

### 2.4 Preparation of Aqueous Extract and Phytochemical Screening

Dried ripened seeds of *C. marianus* were washed thoroughly with plain water and dried in a Tray Dryer (Model No: SE1/2‐44, Germany). After drying of seeds, they were crushed into powder form by a pulverizer (Model No: BE‐100, China). Hundred grams of powdered seeds were taken and mixed with 100 mL of water (eq to 1× aq solution) and kept for maceration for 14 days. After 14 days, the mixture was pressed, and the solution was filtered using a cellulose nitrate filter with a pore size of 8 μm. A 1:100 dilution (referred to as the 1× solution) was prepared by mixing 1 mL of the filtered extract with 99 mL of distilled water. A 2× solution was then prepared by mixing 2 mL of the original filtered extract with 98 mL of distilled water to achieve double the concentration of the 1× solution. Serial dilutions were performed thereafter to obtain up to a 12× dilution, using appropriate dilution factors at each step. 12× aq solution was obtained after 12 dilutions. The aqueous solution of *C. marianus* was subjected to phytochemical screening for alkaloids, glycosides, carbohydrates, phenolics, flavonoids, and saponins using established methods (Shaikh and Patil 2020).

### 2.5 GC–MS Analysis of Extract of *Carduus marianus*

GC–MS analysis was carried out at the Department of Chemistry, Faculty of Science, Forman Christian College, Lahore, using an Agilent GC–MS system, following the method described by (Adebayo et al. 2023) for aqueous plant extracts. The constituents were identified by comparing the obtained spectra and retention times. The results were expressed as the percentage of each component, corresponding to its identified peak, retention time, and molecular weight in the current analysis, as per already published literature of plant aq. extract (Adebayo et al. 2023). These results were further validated by comparison with other published data, the NIST library (MS Interpreter Ver 3.4.5, March 2023 release).

### 2.6 Experimental Design

The experimental design encompassed the investigation of hepatotoxicity. Animals were randomly assigned to six groups (*n* = 8) as follows:

Group I: Normal control—Rats received intraperitoneal (ip) administration of normal saline (1 mL/kg) daily for 3 weeks.

Group II: Negative control—Rats were administered normal saline (1 mL/kg, ip) daily for 3 weeks and an oral dose of paracetamol (750 mg/kg) on days 8 and 16 to induce hepatotoxicity.

Group III: Positive control—Rats received oral administration of silymarin (50 mg/kg) daily for 3 weeks, along with an oral dose of paracetamol (750 mg/kg) on days 8 and 16.

Group IV: Treatment I—Rats were orally administered *C. marianus* aqueous solution 12× (100 mg/kg) daily for 3 weeks, along with an oral dose of paracetamol (750 mg/kg) on days 8 and 16.

Group V: Treatment II—Rats received oral *C. marianus* aqueous solution 12× (200 mg/kg) daily for 3 weeks, combined with an oral dose of paracetamol (750 mg/kg) on days 8 and 16.

Group VI: Treatment III—Rats were treated with oral *C. marianus* aqueous solution 12× (300 mg/kg) daily for three weeks, along with an oral dose of paracetamol (750 mg/kg) on days 8 and 16.

After 24 h, on the 22^nd^ day, each rat was sedated with chloroform (1 mL/kg) before euthanasia. Blood was drawn by cardiac puncture for biochemical marker analysis. Liver samples were collected and preserved in a 10% solution of formalin. Liver tissue weighing 1 g was precisely measured, dissolved in 100 mL of normal saline solution, and homogenized using a homogenizer in a cold‐water bath at approximately 4°C. This 1% homogenate of liver solution was used for analyzing various biochemical markers. The serum was separated from blood by placing the blood contained in an EDTA tube in a centrifuge, operating at 4000 rpm for 20 min. The supernatant was collected and well‐preserved for analysis.

### 2.7 Determination of Biochemical Markers

Serum samples from each rat were analyzed for hepatocellular injury markers aspartate aminotransferase (AST) and alkaline transferase (ALT). The lipid profile, including total lipids, cholesterol, triglycerides, high‐density lipoprotein (HDL), very low‐density lipoprotein (VLDL), and low‐density lipoprotein (LDL) levels, as well as total bilirubin, reflecting liver excretory function, was accessed along with white blood cells (WBCs), albumins, globulins, urea, and creatinine. All analysis was carried out in triplicate using the Beckman Coulter AU 480 auto‐analyzer (Hamburg, Germany).

### 2.8 Estimation of Serum Inflammatory Markers

Inflammatory markers in the serum were evaluated by measuring TNF‐α levels, IL‐6 levels, and NF‐kB levels using spectrophotometric techniques and analyzing kits (Allameh et al. 2023).

### 2.9 Estimation of Oxidative Stress

Liver oxidative stress was evaluated in both serum and tissue samples by measuring enzyme CAT activity, H_2_O_2_ level, and MDA content. These parameters were accessed by using spectrophotometric techniques and analysis kits (Nanjing Jiancheng Bioengineering Institute, China), and the instructions of the manufacturer were followed, while results were expressed in U/mgprot (Gonfa et al. 2023).

### 2.10 Expression of Inflammatory Markers by RT‐ PCR

PCR primers were synthesized to evaluate mRNA expression (Table 1). The PCR reaction was conducted using GoTaq Green master mix, DNA polymerase, and specific primers by using a thermocycler (Applied Biosystems, USA). Amplified products were utilized for electrophoresis on a 2% agarose gel with ethidium bromide and analyzed under ultraviolet (UV) light (Bio Helix Co. Ltd).

**TABLE 1:** Primer sequences of inflammatory mediators.

Primers	Sequence	Gene	Product
Forward Reverse	5’‐AGTCCGGCAGGTCTACTTT‐3′ 5’‐GGAAATTCTGAGCCCGGAGT‐3′	TNF‐alpha	202 bp
Forward Reverse	5’‐TGAGATCCATGCCATTGGCC‐3′ 5’‐AGCTGAGCATGAAGGTGGATG‐3′	NF‐kB	207 bp
Forward Reverse	5’‐CCCACCAAGAACGATAGTCA‐3′ 5’‐CTCCGACTTGTGAAGTGGTA‐3′	IL‐6	247 bp

### 2.11 Histopathological Studies

For histological analysis, liver tissue preserved in formalin solution (10%) was sectioned (5 μm) and stained with Hematoxylin and Eosin (H&E) to assess hepatocyte necrosis, vacuolization, apoptotic bodies, ballooning degeneration, lymphocytic infiltration, and cirrhotic nodules. Morphological alterations were examined in peripheral regions (Gandhi et al. 2023)

### 2.12 Statistical Analysis

Data was expressed as mean ± standard error of the mean (SEM). One‐way analysis of variance (ANOVA) followed by Tukey's multiple‐comparison test was used to analyze intergroup variations. Statistical significance was set at *p* < 0.05. GraphPad Prism software, Version 6.01 (GraphPad Software LLC, San Diego, CA, USA), was utilized for data analysis.

## 3 Results

### 3.1 Phytochemical Analysis

Phytochemical screening of the aqueous seed extract of *C. marianus* indicated the presence of several secondary metabolites, including glycosides, alkaloids, phenolic compounds, flavonoids, and carbohydrates. These findings suggest a diverse phytochemical profile with potential therapeutic relevance.

### 3.2 Characterization of *Carduus marianus* Extract

**FIGURE 1:**
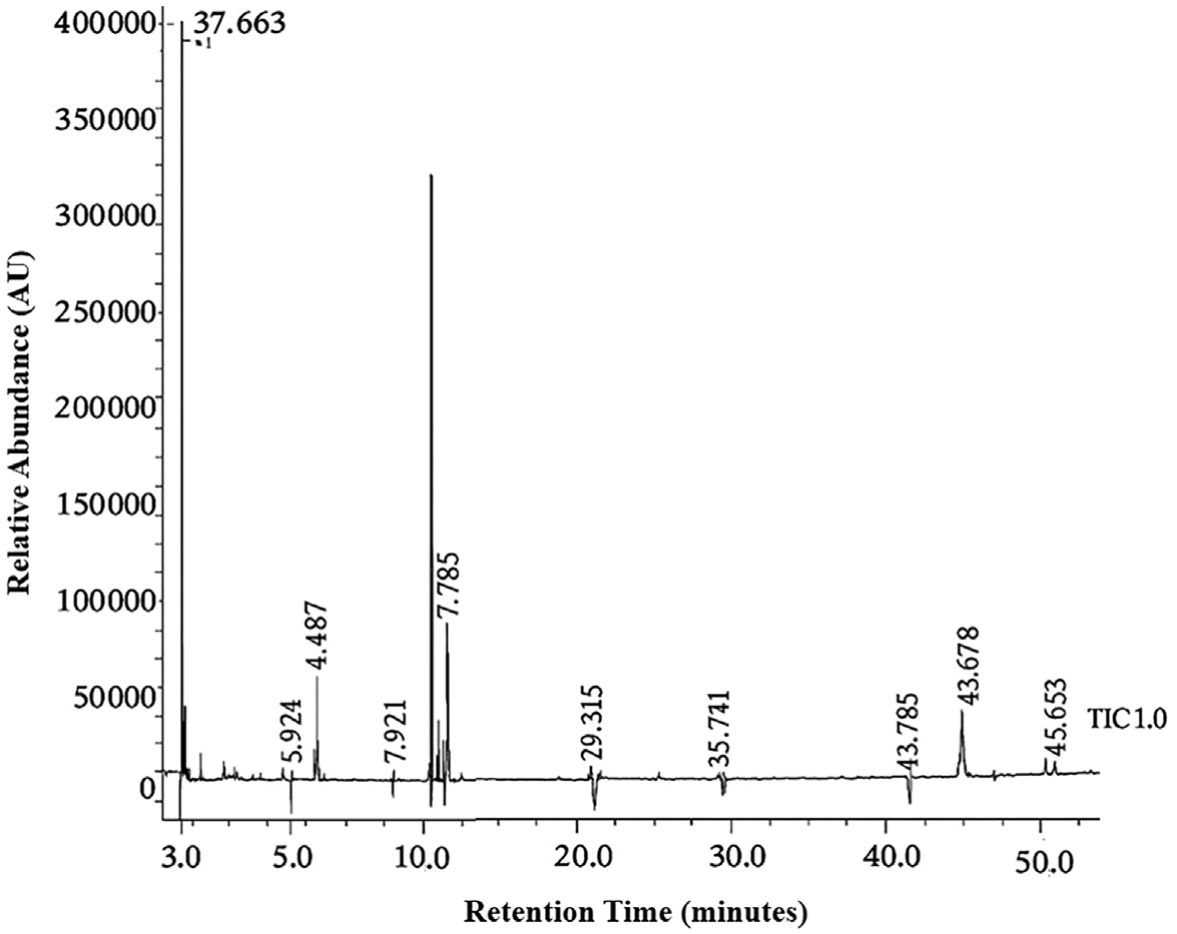
GC–MS chromatogram of bioactive compounds present in *Carduus marianus* extract.

Gas chromatography–mass spectrometry analysis identified twenty constituents, with oleic acid being the major component (62.96%), followed by disiloxane, pentamethyl‐ (5.94%), kauran‐18‐Al, 17‐(acetyloxy)‐, 4 (3.97%), benzene, 1,2‐dimethyl‐ (3.97%), benzene, 1,2‐dimethyl‐ or O‐xylene (3.30%), ethane, 1,1’‐oxybis[2,2‐ dimethoxy‐] (4.51%), beta‐sitosterol (2.91%), and (1.31%) along with other phytochemicals as represented in Figure 1 and Table 2.

**TABLE 2:** Identified bioactive compounds in the aqueous extract of *Carduus marianus* using gas chromatography–mass spectrometry.

Sr. No	RT No	Component name	Molecular formula	Molecular weight	Peak area (%)
1.	2.748	Oleic acid	C_18_H_34_O_2_	282.46	62.96
2.	2.876	Acetic acid, butyl ester	C_6_H_12_O_2_	116	0.62
3.	3.324	Propane, 2,2‐bis(ethylthio)—	C_5_H_14_OSi	118	3.19
4.	3.588	Benzene, ethyl—	C_8_H_10_	106	1.93
5	3.741	Benzene, 1,2‐dimethyl‐ or O‐xylene	C_8_H_10_	106	3.30
6.	3.895	Methyl 3,3‐ dimethoxypropionate	C_5_H_12_O_2_Si	132	0.79
7.	4.164	Benzene, 1,2‐dimethyl—	C_8_H_10_	106	3.97
8.	4.858	Bromobenzene P627	C_6_H_5_Br	156	0.66
9.	6.592	Benzene, (1‐methylethyl)—	C_9_H_12_	120	0.44
10.	7.447	Ethane, 1,1’‐oxybis[2,2‐ dimethoxy—	C_7_H_18_Osi	146	4.51
11.	9.721	1‐Hexanol, 2‐ethyl—	C_8_H_18_O	130	0.25
12.	11.290	Benzene, 1,3‐bis(1‐methylethyl)—	C_12_H_18_	162	0.30
13.	11.496	Disiloxane, pentamethyl—	C_16_H_34_O_5_S_i_	362	5.94
14.	11.851	Benzene, 1,3‐bis(1‐methylethyl)—	C_12_H_18_	162	0.38
15.	12.055	3,5,5‐Trimethyl‐hexanethiol	C_9_H_18_	126	0.16
16.	29.215	1,2‐Benzenedicarboxylic acid, B	C_16_H_22_O_4_	278	0.20
17.	33.574	Phytol	C_20_H_40_O	296	0.72
18.	40.335	1,2‐Benzenedicarboxylic acid, D	C_24_H_38_O	390	0.80
19.	42.678	Kauran‐18‐Al, 17‐(acetyloxy)‐, (4	C_20_H_30_O_3_	318	3.97
20.	44.254	1,1,3,3,5,5,7,7,9,9,11,11,13,13,1 5,15‐Hexa	C_14_H_44_O_6_Si_7_	504	0.25
21.	45.738	1‐Octadecanol	C_15_H_32_O	228	0.17
22.	46.233	Octasiloxane, 1,1,3,3,5,5,7,7,9,9,11,11,13,1	C_29_H_48_O	412	0.25
23.	51.436	Stigmasterol	C_29_H_50_O	414	1.31
24.	52.778	Beta‐sitosterol	C_28_H_48_O	414.7	2.91

*Note:* RT*: Retention time for a single compound to travel through the column. This time is influenced by various factors including the column length, temperature, and carrier gas flow rate.

### 3.3 Effect on Liver Markers

Serum levels of albumin, globulins, ALT, AST, and total bilirubin were assessed to evaluate liver function. Paracetamol‐induced liver damage significantly elevated the levels of specific liver enzymes and total bilirubin compared to the normal control group. However, supplementation with *C. marianus* seeds aq. extract for 3 weeks significantly reduced these levels (*p* < 0.0001) compared to the normal group, indicating a dose‐dependent and effective mitigation of paracetamol‐induced liver toxicity (Table 3) 2.3 ± 0.45.

**TABLE 3:** Effects of *Carduus marianus* on liver function tests.

Serum profile	LFTs				
	Total bilirubin (mg/dl)	ALT (U/L)	AST (U/L)	Albumin (g/dl)	Globulins (g/dl)
Normal control (normal saline 1 ml/kg, ip)	3.22 ± 0.19	74.6 ± 10.09	149.8 ± 9.60	3.24 ± 0.23	2.49 ± 0.22
Negative control (normal saline 1 mL/kg + paracetamol 750 mg/kg)	7.82 ± 0.88	161.4 ± 38.74	291 ± 6.44	4.36 ± 0.36	3.18 ± 0.44
Positive control (Silymarin 50 mg/kg + normal saline 1 mL/kg)	4.24 ± 0.27	89.56 ± 2.84	208.8 ± 12.68	2.3 ± 0.45	1.28 ± 0.39
Treatment I ( *C. marianus* 12 × 100 mg/kg + paracetamol 750 mg/kg)	4.44 ± 0.46*	50.6 ± 8.53*	152.8 ± 9.12*	3.38 ± 0.44*	2.54 ± 0.27**
Treatment II ( *C. marianus* 12 × 200 mg/kg + paracetamol 750 mg/kg)	4.32 ± 0.33***	48.4 ± 7.33**	149.6 ± 8.76**	3.28 ± 0.18**	2.52 ± 0.45***
Treatment III ( *C. marianus* 12 × 300 mg/kg + paracetamol 750 mg/kg)	4.18 ± 0.18****	45 ± 2.92****	149.6 ± 4.34****	3.26 ± 0.44***	2.4 ± 0.29****

*Note:* Results are presented as mean ± SEM for eight animals per group. Treatment Groups I to III received *Carduus marianus* 12× aqueous solution at different doses and paracetamol (750 mg/kg) on Days 8th and 16th, administered as 100, 200, and 300 mg/kg per 150 ± 10 g body weight. Statistical significance compared to the negative control group is indicated as follows: ****(*p* < 0.0001), ***(*p* < 0.001), **(*p* < 0.01), and *(*p* < 0.05).

### 3.4 Effect on Lipid Profile

Significant increases were observed in cholesterol, total lipids, triglycerides, VLDL, and LDL levels, while HDL levels decreased in paracetamol‐treated rats compared to the negative control group. Supplementation with *C. marianus* significantly decreased these parameters (*p* < 0.0001) and increased HDL levels (*p* < 0.0001), as shown in Table 4.

**TABLE 4:** Effects of *Carduus marianus* on lipid profile.

Serum profile	Lipid profile					
	Total lipids (mg/dl)	Cholesterol (mg/dl)	Triglycerides (mg/dl)	HDL (mg/dl)	LDL (mg/dl)	VLDL (mg/dl)
Normal control (normal saline 1 mL/kg, ip)	350.8 ± 4.44	60.2 ± 1.92	74.6 ± 3.05	43.6 ± 2.70	64.6 ± 3.05	13.2 ± 2.77
Negative control (normal saline 1 mL/kg + paracetamol 750 mg/kg)	548 ± 40.12	107.4 ± 14.08	147 ± 27.49	29.6 ± 5.81	90.8 ± 7.82	61 ± 14.87
Positive control (Silymarin 50 mg/kg + normal saline 1 mL/kg)	400.8 ± 16.93	70.2 ± 13.92	87.4 ± 3.78	79.2 ± 6.38	72.6 ± 8.41	25.6 ± 8.05
Treatment I ( *Carduus marianus* 12 × 100 mg/kg + paracetamol 750 mg/kg)	392.6 ± 31.8*	70 ± 14.73*	89.6 ± 7.20*	51.8 ± 6.53**	71.6 ± 11.95**	21 ± 4.85**
Treatment II ( *Carduus marianus* 12 × 200 mg/kg + paracetamol 750 mg/kg)	375 ± 30.55**	67 ± 13.02**	85.6 ± 10.78**	50.8 ± 7.50**	69.4 ± 5.73***	18.6 ± 1.52**
Treatment III ( *Carduus marianus* 12 × 300 mg/kg + paracetamol 750 mg/kg)	349.6 ± 27.98****	64.6 ± 13.90****	80.6 ± 10.36****	47.4 ± 4.72****	65.8 ± 4.32****	17.8 ± 0.84****

*Note:* Results are presented as mean ± SEM for eight animals per group. Treatment Groups I to III received *Carduus marianus* 12× aqueous solution at different doses and paracetamol (750 mg/kg) on days 8^th^ and 16^th^, administered as 100, 200, and 300 mg/kg per 150 ± 10 g body weight. Statistical significance compared to the negative control group is indicated as follows: ****(*p* < 0.0001), ***(*p* < 0.001), **(*p* < 0.01), and *(*p* < 0.05).

### 3.5 Effect on Renal Function Markers and WBC Count

Paracetamol‐induced liver damage significantly increased serum levels of urea, creatinine, and WBC count compared to the negative control group. However, supplementation with *C. marianus* seeds aq. extract for 3 weeks significantly lowered these renal markers (*p* < 0.0001) and WBC count (*p* < 0.0001), indicating its protective effect against paracetamol‐induced liver toxicity in treatment rats (Table 5).

**TABLE 5:** Effects of *Carduus marianus* on renal profile and WBC count.

Serum profile	RFTs		WBC count
	Urea (mg/dl)	Creatinine (mg/dl)	(×10^3^/μL)
Normal control (normal saline 1 ml/kg, ip)	57.4 ± 4.51	0.42 ± 0.08	6.41 ± 1.03
Negative control (normal saline 1 mL/kg + paracetamol 750 mg/kg)	94 ± 6.20	0.88 ± 0.04	13.71 ± 0.52
Positive control (Silymarin 50 mg/kg + normal saline 1 mL/kg)	71.6 ± 3.78	0.62 ± 0.15	10.87 ± 0.39
Treatment I ( *Carduus marianus* 12 × 100 mg/kg + paracetamol 750 mg/kg)	57.8 ± 1.79**	0.45 ± 0.03*	8.79 ± 0.47***
Treatment II ( *Carduus marianus* 12 × 200 mg/kg + paracetamol 750 mg/kg)	57 ± 4.53****	0.446 ± 0.05**	7.73 ± 0.47****
Treatment III ( *Carduus marianus* 12 × 300 mg/kg + paracetamol 750 mg/kg)	56.8 ± 5.36****	0.422 ± 0.06****	6.65 ± 0.43****

*Note:* Results are presented as ± SEM for eight animals per group. Treatments I–III involved *Carduus marianus* 12× aqueous solution at different doses and paracetamol at 750 mg/kg administered on days 8^th^ and 16^th^, specifically at doses of 100, 200, and 300 mg/kg per 150 ± 10 g. The symbols ****, ***, **, and *indicate statistical significance compared to the negative control group, with *p* < 0.0001, < 0.001, < 0.01, and < 0.05, respectively.

### 3.6 Effect on Inflammatory Markers

Paracetamol treatment significantly enhanced NF‐kB, TNF‐α, and IL‐6 activity in rats (*p* < 0.0001), while supplementation with *C. marianus* significantly reduced the levels of these inflammatory markers (*p* < 0.0001) compared to paracetamol‐treated rats (Table 6 and Figure S1).

**TABLE 6:** Effects of *Carduus marianus* on inflammatory markers.

Serum profile	Inflammatory markers		
	TNF‐alpha (pg/ml)	IL‐6 (pg/ml)	NF‐kB (U/L)
Normal control (normal saline 1 ml/kg, ip)	2.19 ± 0.056	78.70 ± 8.39	33.34 ± 4.427
Negative control (normal saline 1 mL/kg + paracetamol 750 mg/kg)	5.33 ± 0.228	115.84 ± 5.31	58.3 ± 7.76
Positive control (Silymarin 50 mg/kg + normal saline 1 mL/kg)	2.4 ± 0.308	87.3 ± 1.83	43.64 ± 6.59
Treatment I ( *Carduus marianus* 12 × 100 mg/kg + paracetamol 750 mg/kg)	2.6 ± 0.374**	81.8 ± 3.56*	40.6 ± 2.3**
Treatment II ( *Carduus marianus* 12 × 200 mg/kg + paracetamol 750 mg/kg)	0.28 ± 0.29****	79.08 ± 1.8****	37.25 ± 6.63****
Treatment III ( *Carduus marianus* 12 × 300 mg/kg + paracetamol 750 mg/kg)	2.18 ± 0.259****	78.4 ± 2.608****	35.8 ± 4.51****

*Note:* Treatments I–III involved *Carduus marianus* 12× aqueous solution at different doses and paracetamol at 750 mg/kg administered on days 8^th^ and 16^th^, specifically at doses of 100, 200, and 300 mg/kg per 150 ± 10 g. The symbols ****, ***, **, and *indicate statistical significance compared to the negative control group, with *p* < 0.0001, < 0.001, < 0.01, and < 0.05, respectively.

### 3.7 Effect on Antioxidant Enzymes

In paracetamol‐treated rats, CAT activity was lower, and MDA and H_2_O_2_ activity were higher than those observed in normal rats, indicating severe cellular damage. However, administration of *C. marianus* significantly increased CAT levels and reduced MDA and H_2_O_2_ levels (*p* < 0.0001), indicating hepatocyte survival (Table 7).

**TABLE 7:** Effects of *Carduus marianus* on oxidative stress.

Serum profile	Oxidative stress markers					
	CAT		H_2_O_2_		MDA	
	Serum (U/mL)	Liver tissue (U/mgprot)	Serum (U/mL)	Liver tissue (U/mgprot)	Serum (U/mL)	Liver tissue (U/mgprot)
Normal control (normal saline 1 ml/kg, ip)	45.084 ± 3.789	40.8 ± 2.83	6.09 ± 3.67	4.42 ± 1.02	7.13 ± 0.752	4.81 ± 0.587
Negative control (normal saline 1 mL/kg + paracetamol 750 mg/kg)	36.89 ± 3.25	29.2 ± 2.8	21.7 ± 3.27	17.6 ± 3.45	14.48 ± 2.517	9.71 ± 1.18
Positive control (Silymarin 50 mg/kg + normal saline 1 mL/kg)	54.14 ± 2.882	62.22 ± 2.31	7.2 ± 1.39	7.56 ± 1.2	10.31 ± 0.46	6.04 ± 1.519
Treatment I ( *Carduus marianus* 12 × 100 mg/kg + paracetamol 750 mg/kg)	49.9 ± 4.2*	49.9 ± 3.4**	15.1 ± 5.35*	8.4 ± 0.78****	8.98 ± 0.56**	5.20 ± 0.84***
Treatment II ( *Carduus marianus* 12 × 200 mg/kg + paracetamol 750 mg/kg)	51.9 ± 2.7*	50.3 ± 5.8****	6.01 ± 2.68****	11.12 ± 1.1****	7.96 ± 1.377**	4.88 ± 0.47****
Treatment III ( *Carduus marianus* 12 × 300 mg/kg + paracetamol 750 mg/kg)	52.59 ± 2.17**	51.2 ± 3.23****	3.54 ± 2.207****	7.68 ± 1.46****	7.64 ± 1.096****	4.45 ± 0.332****

*Note:* Treatments I–III involved *Carduus marianus* 12× aqueous solution at different doses and paracetamol at 750 mg/kg administered on days 8^th^ and 16^th^, specifically at doses of 100, 200, and 300 mg/kg per 150 ± 10 g. The symbols ****, ***, **, and *indicate statistical significance compared to the negative control group, with *p* < 0.0001, < 0.001, < 0.01, and < 0.05, respectively.

### 3.8 Histopathology of Liver

**FIGURE 2:**
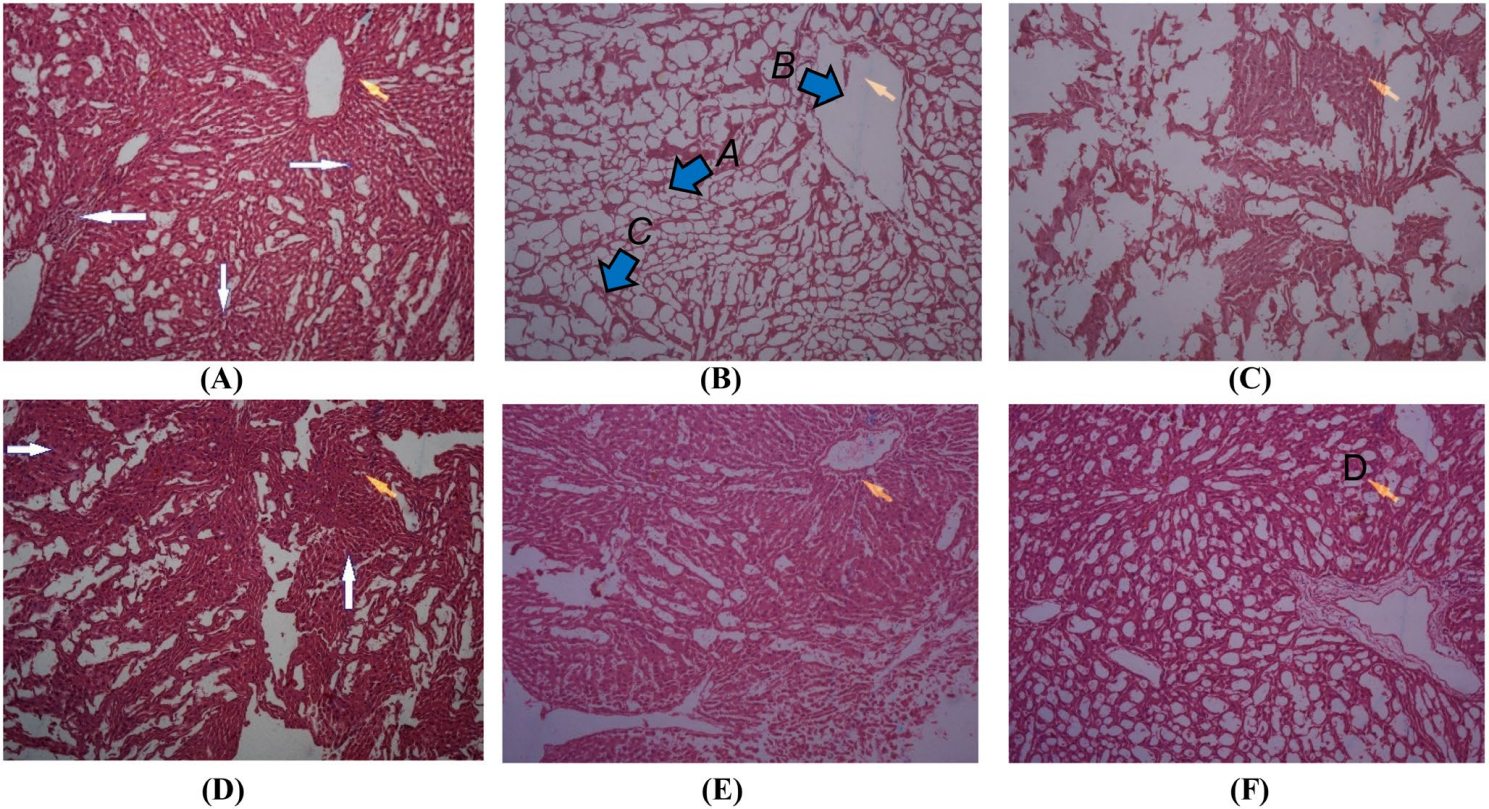
Histopathological analysis of liver tissue (A) normal group, (B) negative control, (C) positive group, (D) Treatment I ( *C. marianus* , 100 mg/kg), (E) Treatment II ( *C. marianus* , 200 mg/kg), and (F) Treatment III ( *C. marianus* , 300 mg/kg) treated in rats for 21 days. A enlarged hepatocytes, B fat droplets, C binucleation, and D multinucleated.

Normal liver morphology was observed in the control group, while paracetamol‐treated rats exhibited sporadic enlarged hepatocytes, fatty degeneration, and multinucleated hepatocytes. Rats treated with *C. marianus* showed a more typical appearance with multinucleated hepatocytes, indicating cellular regeneration and protection against paracetamol‐induced liver damage. No signs of cholestasis, necrosis, bleeding, or inflammation were observed in rats treated with *C. marianus* (Figure 2).

## 4 Discussion

Liver diseases significantly impact global mortality, with approximately 2 million deaths annually attributed to complications such as cirrhosis and hepatocellular carcinoma, collectively accounting for 3.5% of global deaths. Maintaining liver health is essential for normal physiological functions, and disruptions can lead to severe consequences (Asrani et al. 2019). A healthy immune system plays a crucial role in overall health by defending against pathogens and regulating inflammatory responses. However, exposure to environmental toxins and medications can impair immune function, resulting in immune‐related disorders. Phytochemicals, naturally occurring compounds found in plants, have gained attention for their immunomodulatory properties, which support immune system function (Behl et al. 2021).

Normal immunological and physiological processes, as well as the internal environment, depend critically on the host body's immune system functioning properly. To prevent hypersensitive reactions and immune‐related disorders, a healthy immune system bolsters the body's resistance against diseases, infections, and undesirable microbes. Corrective interaction between acquired immune system components and innate immune cells produces optimal immunological responses. The immune system has garnered more attention recently as a major target of toxicity brought on by exposure to chemicals, medications, and environmental contaminants. Phytochemicals are naturally occurring compounds that are crucial in regulating immunological responses that are favorable (Behl et al. 2021).

Hepatoprotection has been observed for several plants, including *Andrographis paniculata* , *Bauhinia purpurea* , *Commelina nudiflora* , *Dillenia suffruticosa* , *Elaeis guineensis* , *Lygodium microphyllum* , and *Nephrolepis biserrata* . Furthermore, because these plants include a variety of phytochemicals, such as alkaloids, saponins, flavonoids, tannins, terpenoids, steroids, polyphenols, and diterpenoid lactones, it has been demonstrated that they are essential in reducing cellular damage. These plants contain various compounds that have been shown to have anti‐inflammatory, hepatoprotective, andrographolide, rosmarinic acid, phenol, eugenol, 9,12‐octadecadienoic, n‐hexadecanoic acid, dihydroxy dimethoxy flavone, sitosterol, desmethoxycurcumin, quercetin, linoleic acid, stigmasterol, kojic acid, indole‐2‐one, α‐terpinol, linalool, kaempferol, catechin, ellagic acid, and oleanolic acid properties (Venmathi Maran et al. 2022).

A common, widely accessible analgesic and antipyretic medication that has been used for decades and is safe and effective at therapeutic levels is paracetamol (in Europe and the rest of the globe) or N‐acetyl‐para‐aminophenol (APAP) in the US and Japan. However, an excessive amount of APAP can cause serious liver damage, which can lead to acute liver failure (ALF) and even (Jaeschke and Ramachandran 2024). Excessive NAPQI is generated in the event of an APAP overdose or prolonged use, which reduces the amount of GSH stored in the cytoplasm and modifies the electron transport chain (ETC) in the mitochondria, resulting in endoplasmic reticulum (ER) and oxidative stress, which finally results in hepatocyte mortality (Hionides‐Gutierrez et al. 2024).

There are several treatment options available for liver impairment induced by synthetic medications, although many are associated with adverse side effects. Therefore, this study aimed to explore the potential for a natural, food‐derived substitute to guard against long‐term liver damage, which is a significant cause of chronic illnesses of the liver. The present study was focused on the use of phytochemicals for benefiting liver health extracted from *C. marianus* aqueous solution. Phytochemicals are compounds that have active roles in diets based on plants. Due to mammal–plant coevolution and adaptation, phytochemicals present in foods such as fruits, vegetables, grains, and seed oils are regarded as generally safe for ingestion. Numerous illnesses affecting people are linked to oxidative stress, which can be brought on by environmental pollutants found in food, water, and the air. Dietary choices and lifestyle choices like smoking and inactivity also have a significant role in the development of disease in people. Plant‐based diets typically contain phenolic acids, flavonoids, and carotenoids, which have potent antioxidant qualities. As a result, they eliminate the body's excess active oxygen and shield cells from harm, lowering the risk of Alzheimer and cardiovascular disease. Obesity is typically caused by food and inactivity; plant‐based diets alter lipid composition and metabolism, lowering the risks associated with obesity (Guan et al. 2021).

Drug‐induced liver injury (DILI) is a leading cause of acute liver damage. Among the various hepatotoxic agents, acetaminophen (also known as paracetamol) is a commonly used antipyretic and analgesic that is well documented to induce liver injury when consumed in excessive doses (Jaeschke et al. 2020; Rotundo and Pyrsopoulos 2020). Paracetamol (APAP), a widely recognized antipyretic and nonsteroidal anti‐inflammatory drug (NSAID), is generally safe at therapeutic doses but can cause severe hepatotoxicity in cases of overdose (Begriche et al. 2023; Chilvery et al. 2023). In this study, we evaluated the hepatoprotective potential of *C. marianus* in a rat model of paracetamol‐induced liver injury.

Liver enzymes ALT, AST, alkaline phosphatase, and bilirubin are essential for the proper functioning of hepatocytes. Paracetamol administration increased enzyme and bilirubin levels from damaged hepatocytes while decreasing albumin and globulin levels. In contrast, supplementation with *C. marianus* significantly mitigated these adverse effects, demonstrating its potential to counteract paracetamol‐induced liver toxicity (Awan et al. 2020). These results align with previous studies that have reported the hepatoprotective effects of *C. marianus* in various models of liver injury (Kandasamy and Mathew 2021). For instance, the antioxidant properties of silymarin, a key bioactive compound in milk thistle, are well‐documented for their protective effects against drug‐induced liver injury (Adetuyi et al. 2021).

The antioxidant properties of *C. marianus* were evaluated, showing promising results in reducing oxidative stress markers such as malondialdehyde (MDA) and hydrogen peroxide (H_2_O_2_), while enhancing Catalase (CAT) activity. Oxidative stress plays a critical role in liver injury by damaging cellular components, leading to inflammation and cell death. Our findings suggest that the antioxidant activity of *C. marianus* contributes to the preservation of hepatocyte integrity and function. This is consistent with studies on other hepatoprotective agents. For instance, a study by (Aghemo et al. 2022) demonstrated that silymarin significantly reduces oxidative stress markers in various liver disease models (Begriche et al. 2023). In 2022, Jang demonstrated that the well‐known flavonol glycoside hyperoside (Hyp), also called quercetin‐3‐O‐galactoside or 3‐O‐β‐D‐galactopyranosyl, is found in large quantities in a variety of fruits, vegetables, and medicinal plants (Jang 2022). Numerous biological activities have been proposed for Hyp, including hepatocellular antioxidant defense through the activation of erythroid‐related nuclear factor 2. Another study conducted by Abdelghffar et al. (2022) in mice demonstrated thyme (*Thymus fontanesii*), the plant's protective and advantageous properties against CCl_4_‐induced liver damage (Abdelghffar et al. 2022). Similarly, a study by Sun et al. (2022) demonstrated that the extract of *Jasminum grandiflorum* L. (JG) significantly alleviated inflammation and oxidative stress in the liver (Sun et al. 2022).

Inflammatory markers such as TNF‐α, IL‐6, and NF‐kB were elevated following paracetamol administration, indicating increased inflammation. Treatment with *C. marianus* significantly reduced these inflammatory markers, suggesting its anti‐inflammatory effects, possibly mediated by its oleic and linoleic acid content (Santamarina et al. 2021). These findings are supported by studies showing that phytochemicals can modulate inflammatory pathways. El‐Kot et al. (2023) demonstrated the anti‐inflammatory effects of silymarin, showing its ability to reduce the expression of pro‐inflammatory cytokines in liver cells (El‐Kot et al. 2023). ur Rahman et al. (2022) demonstrated that mice intoxicated with paracetamol experienced hepatoprotective effects from walnut oil and *C. tuberculata* (ur Rahman et al. 2022). Similarly, Putera et al. (2023) revealed that the ethyl acetate leaf extract of *D. stramonium* (DSL‐EA) exhibited significant anti‐inflammatory potential (Putera et al. 2023).

The hepatoprotective effects of *C. marianus* can be attributed to its multifaceted action involving antioxidant, anti‐inflammatory, and immunomodulatory mechanisms. Our study's findings on the reduction of liver enzymes, bilirubin, lipid profile markers, and oxidative stress parameters corroborate with existing literature on the hepatoprotective potential of phytochemicals. Research conducted by Loguercio and Festi (2011) demonstrated that silymarin, a flavonoid derived from milk thistle, significantly reduces liver enzyme levels and mitigates oxidative stress in patients with liver disease (Loguercio and Festi 2011). Moreover, the anti‐inflammatory properties of *C. marianus* are particularly relevant in the context of drug‐induced liver injury, where inflammation plays a central role in the pathogenesis. The significant reduction in TNF‐α, IL‐6, and NF‐kB levels observed in our study aligns with the findings of El‐Kot et al. (2023), who reported that silymarin reduces the expression of these pro‐inflammatory cytokines in various models of liver injury (El‐Kot et al. 2023).

Numerous studies have investigated the use of food‐derived ingredients in the management of inflammation. Oleic acid and other unsaturated fatty acids are among the substances that may have an impact on inflammation. Oleic acid affects gene expression, intracellular signaling cascades, receptors, and the fluidity of cell membranes. It is possible that oleic acid directly controls the production and function of antioxidant enzymes. The activation of anti‐inflammatory ones and suppression of proinflammatory cytokines may be linked to the anti‐inflammatory impact. Oleic acid is highlighted as a natural activator of sirtuin 1 (SIRT1) in the mechanism that has been well described. Derived from oleic acid, oleoyl ethanolamide (OEA) is an endogenous ligand of the nuclear receptor peroxisome proliferator‐activated receptor alpha (PPARα). Energy and dietary fat intake are controlled by OEA. Due to its ability to regulate mRNA expression homeostasis and produce positive anti‐inflammatory effects, oleic acid has been proposed as a possible therapeutic drug (Santa‐María et al. 2023). Under the conditions of both palmitic acid (PA) and endoplasmic reticulum stress (ER) inducer‐induced lipotoxicity, oleic acid can effectively improve autophagy dysfunction. Additionally, oleic acid‐mediated regulation of lysosome dysfunction through TFEB plays a significant role, indicating that the regulation of the ER stress–autophagy axis is a crucial mechanism in OA‐driven protection in NAFLD (Liu et al. 2023).

For millennia, people have recognized the health and well‐being benefits of phytochemicals. These naturally occurring chemical compounds in plants are known for their disease‐preventing or protective properties. Phytochemical screening has identified important bioactive substances such as flavonoids, phenols, alkaloids, polysaccharides, and glycosides. Since these bioactive secondary metabolites are strongly linked to considerable antioxidant activity, the presence of carbohydrates, phenolic compounds, glycosides, flavonoids, and alkaloids may account for the high free radical scavenging activity seen by *C. marianus* (Bakoyiannis et al. 2019; Kaushik et al. 2021; Shoker 2020).

## 5 Conclusions

Our study demonstrated that *C. marianus* has hepatoprotective properties in a rat model of hepatotoxicity triggered by paracetamol. These outcomes may be related to the plant's anti‐inflammatory and antioxidant potential. The importance of *C. marianus* in liver health research is reinforced by these results, which are in line with earlier findings. Given the promising results of our studies, future investigations could focus on the detailed molecular pathways through which *C. marianus* exerts its protective effects, potentially leading to the development of novel therapeutic agents for liver diseases.

## Author Contributions

**Mehreen Nasir:** data curation (equal), investigation (lead), writing – original draft (equal). **Faisal Gulzar:** conceptualization (lead), supervision (lead). **Sajida Jamil:** data curation (equal), investigation (equal), software (equal). **Irfan Anjum:** drafting and resources (equal). **Abdul Malik** and **Suhail Akhtar:** funding acquisition (equal). **Nouman Ali:** resources (equal), software (equal).

## Ethics Statement

The protocols for using rats and collecting samples in the current study were approved by the Institutional Research Ethics Committee of the University of Lahore, Lahore (Certification No. IREC‐2023‐48, certification date on 20 June 2023).

## Consent

The authors have nothing to report.

## Conflicts of Interest

The authors declare no conflicts of interest.

## Supporting information


**Figure S1.** Effect of *C. marianus* on the mRNA expression levels of IL‐6, NF‐KB, and TNF‐alpha in paracetamol‐induced hepatotoxicity.

## Data Availability

The data that support the findings of this study are available from the corresponding author upon reasonable request.
