# Supplementary figures and images for: Evaluation of Nutraceutical Potential of Carduus marianus : Antioxidant and Hepatoprotective Effects in Paracetamol‐Induced Hepatotoxicity and GC–MS Analysis
Source: Food Sci Nutr. 2025 Jul 18;13(7):e70474. doi: 10.1002/fsn3.70474 (PMC12272808; doi:10.1002/fsn3.70474)

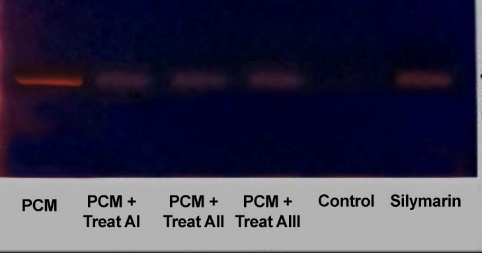

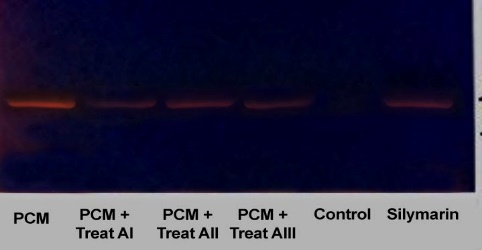

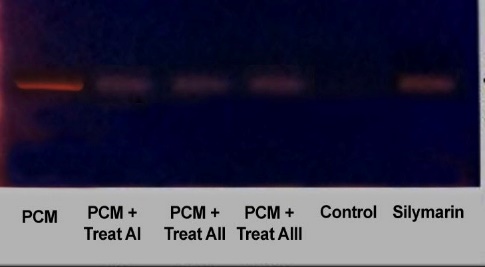


(A) (B) (C)

***Supplementary* Figure 1.** PCR analysis of inflammatory biomarkers IL-6 (A), NF-kβ (B) and TNF-α (C).

Supplement: Supplementary file 1 — Figure S1. Effect of C. marianus on the mRNA expression levels of IL‐6, NF‐KB, and TNF‐alpha in paracetamol‐induced hepatotoxicity. [file FSN3-13-e70474-s001.docx]
